# Life Cycle Assessment of Sustainable Asphalt Pavement Solutions Involving Recycled Aggregates and Polymers

**DOI:** 10.3390/ma14143867

**Published:** 2021-07-11

**Authors:** Cristina Oreto, Francesca Russo, Rosa Veropalumbo, Nunzio Viscione, Salvatore Antonio Biancardo, Gianluca Dell’Acqua

**Affiliations:** Department of Civil, Construction and Environmental Engineering, University of Naples Federico II, Via Claudio 21, 80125 Naples, Italy; cristina.oreto@unina.it (C.O.); francesca.russo2@unina.it (F.R.); nunzio.viscione@unina.it (N.V.); salvatoreantonio.biancardo@unina.it (S.A.B.); gianluca.dellacqua@unina.it (G.D.)

**Keywords:** asphalt mixtures, waste recycling, life cycle inventory, life cycle impact assessment, cold in-place recycling, pavement design

## Abstract

The pursuit of sustainability in the field of road asphalt pavements calls for effective decision-making strategies, referring to both the technical and environmental sustainability of the solutions. This study aims to compare the life cycle impacts of several pavement solution alternatives involving, in the binder and base layers, some eco-designed, hot- and cold-produced asphalt mixtures made up of recycled aggregates in substitution for natural filler and commercial recycled polymer pellets for dry mixture modification. The first step focused on the technical and environmental compatibility assessment of the construction and demolition waste (CDW), jet grouting waste (JGW), fly ash (FA), and reclaimed asphalt pavement (RAP). Then, three non-traditional mixtures were designed for the binder layer and three for the base layer and characterized in terms of the stiffness modulus. Asphalt pavement design allowed for the definition of the functional units of Life Cycle Assessment (LCA), which was applied to all of the pavement configurations under analysis in a “from cradle to grave” approach. The LCA results showed that the best performance was reached for the solutions involving a cold, in-place recycled mixture made up of RAP and JGW in the base layer, which lowered all the impact category indicators by 31% on average compared to those of the traditional pavement solution. Further considerations highlighted that the combination of a cold base layer with a hot asphalt mixture made up of CDW or FA in the binder layer also maximized the service life of the pavement solution, providing the best synergistic effect.

## 1. Introduction

In the last few years, international efforts have been made to develop a list of sustainable development goals [1] that also involve industries and infrastructures, aiming to increase resource-use efficiency and greater adoption of clean and environmentally sound technologies to achieve a more sustainable future for all.

Pavement engineers have pursued the idea of sustainable infrastructures through the adoption of, on the one hand, innovative asphalt materials made up of waste in substitution of natural aggregates and, on the other hand, of alternative production technologies, primarily focusing on reducing the asphalt mixture production temperature from 170–180 °C to 50–60 °C, saving energy and fossil fuels, and extending the service life of road pavements.

Several researchers experimented with the introduction of waste originated from different sources into asphalt mixtures, such as construction and demolition waste (CDW) [2,3], ceramic waste [4,5], waste steel [6], reclaimed asphalt pavement (RAP) [7], plastic waste [8,9], several biomaterials [10,11], and industrial by-products [12,13]. The main goal of these laboratory investigations was to obtain eco-friendly asphalt mixtures that were also sustainable in terms of mechanical properties and durability, so as to minimize maintenance treatments and achieve cost-effective asphalt pavements in the long term.

For example, Choudhary et al. [14] evaluated the mechanical properties of hot asphalt mixtures containing carbide lime, which is a by-product of acetylene production, as a filler. Specifically, the hot asphalt mixture with 7% carbide lime was compared with the traditional mixture with the same percentage of the limestone filler in terms of the indirect tensile strength (+7%), the moisture sensitivity (−4%), and the stiffness modulus at a 35 °C test temperature (+3%). Similarly, Simone et al. [15] evaluated the beneficial effects of 7% waste glass powder into hot asphalt mixtures in terms of stiffness modulus at 5, 20, and 35 °C; they found out that the asphalt mixture with waste glass powder achieved 9 and 0.2% higher stiffness modulus at 5 and 20 °C, respectively, compared to that of traditional hot asphalt mixture with 7% limestone filler. At 35 °C, on the contrary, the stiffness modulus was lowered by 8% for the mixture made up of waste glass powder.

Other studies focused on the sustainable modification of asphalt binders to mainly improve the stiffness and the self-healing properties of asphalt mixtures using, for example, waste cooking oil [16,17], waste toner [18], or waste tire rubber [19]. Some researchers, like Wang et al. [20], added 0.4% recycled basalt fibers with a length of 6 mm to a hot asphalt mixture; they tested the stiffness modulus of the mentioned fiber-modified mixture and a traditional hot asphalt mixture after 15 freeze–thaw cycles. The fiber-modified mixture showed 10% lower damage degree at the end of the freeze–thaw cycles compared to the traditional hot asphalt mixture, always maintaining the stiffness modulus above 2500 MPa.

In order to maximize the environmental benefits of waste recycling into asphalt mixtures, a cold recycling mix design has been investigated in the literature [21,22]. In cold asphalt mixtures, RAP aggregates, eventually combined with more virgin or waste aggregates to comply with specific size distribution requirements, are bonded together with bitumen emulsion or foamed bitumen to enhance the workability and compactability of the cold asphalt mixture at a low mixing temperature. Moreover, a small percentage of cement is often added to the mixture to accelerate the emulsion breaking by consuming the water via hydration and enhance the mechanical performance of cold asphalt mixtures [23]. Several researchers demonstrated that the cold production of asphalt mixtures, other than avoiding the disposal of large quantities of old milled pavements in landfills, does not seriously affect their mechanical performance. For example, Lin et al. [24] compared the stiffness modulus of a cold recycled asphalt mixture (100% RAP, 3.5% asphalt emulsion, 3.8% water, and 2% cement by the weight of RAP aggregates) to that of a traditional hot mix asphalt (100% limestone aggregates and 3.9% bitumen by the weight of aggregates). The cold asphalt mixture resulted in being stiffer than the hot one, showing, on average, a 200% higher stiffness modulus in the high-temperature range compared to that of the hot asphalt mixture.

The improvement of the material’s mechanical performance, the reduction of waste in landfills, natural aggregates’ extraction, and the energy requirement to produce, build, and maintain asphalt pavements are themselves indicators of the environmental sustainability of a solution. Anyhow, the quantitative analysis of the environmental impacts of pavement systems along the entire life cycle, known as the Life Cycle Assessment (LCA) procedure, is a key element to prevent the shifting of negative impacts on the environment from one stage of the life cycle to another and help decision makers to compare multiple solutions and select the one that has the lowest impact on the environment. As already pointed out by Watson et al. [25], asphalt mixture production highly contributes to greenhouse gas emissions due to the high production temperature; therefore, a careful pavement design phase is required to minimize fuel, energy, and raw materials consumption throughout the life cycle [26], during both initial construction and maintenance phases [27].

Several LCA analyses have been carried out on innovative road materials [28,29] and maintenance techniques [30,31] to estimate the environmental compatibility of previously eco-designed asphalt materials and compare them to traditional hot mix asphalt solutions. Some authors focused on the material production phases, highlighting that the increase of the amount of recycled material in the asphalt mixture most certainly entails a decrease in the environmental impacts [32].

For example, Zhang et al. [33] applied the LCA methodology to evaluate the life cycle impacts of a bio-oil-modified asphalt and compared them to those of a traditional hot asphalt mixture. They found that the greenhouse gas emission in the bio-asphalt production process was lower (−38%, −8%, and −7% of CH_4_, CO_2_, and NO_x_ emissions, respectively) than that of neat bitumen’s production. Besides, the energy consumption of bio-asphalt mixture was 13% lower than that of conventional asphalt mixture during asphalt mixture production, laying, and compacting operations. In another study, Praticò et al. [34] evaluated the environmental impacts of a road-pavement-wearing course made up of warm-mix asphalt (WMA), an asphalt mixture produced at a temperature of 140–150 °C, with 45% RAP used in substitution for coarse aggregates, according to the ILCD midpoint impact indicators and the global energy requirement (GER) and compared them to those of a traditional hot asphalt mixture. The results showed that the combination of WMA and RAP allows the consumption of energy, virgin bitumen, and aggregates to be reduced, causing a reduction equal to 19, 14, and 8% of the GER, climate change, and acidification potential indicators, respectively, compared to those of a traditional wearing course.

Others, such as Diaz-Piloneta et al. [35], combined the laboratory testing and mix design phase with the LCA of an asphalt mixture containing blast oxygen furnace slag; they pointed out that slag aggregates had better physical properties than the natural aggregates, as well as better rutting and fatigue cracking resistance of the resulting mixture with 15% slag aggregates compared to the traditional one. However, the application of LCA methodology in a “from cradle to gate” approach highlighted that the transportation distance of the natural and recycled aggregates to the asphalt plant highly influenced greenhouse gas emissions. In particular, the blast furnace oxygen slag should be considered environmentally feasible for transportation distances lower than 200 km, beyond which natural aggregates should be preferred.

In this context, although numerous studies have proved the mechanical and environmental effectiveness of using different waste to enhance the sustainability of asphalt mixtures, they focus mainly on the evaluation of the environmental performance of specific eco-designed materials involving one, or at most two, types of waste. Moreover, the comparison of the environmental impacts of the studied materials with newly designed asphalt mixtures is difficult and, in most cases, even impossible, due to the strong dependence of LCA results from the geographic conditions (e.g., the position of the production facilities, the origin of waste, and transportation distances) and the boundary assumed for the system under analysis (i.e., the processes included in the analysis).

## 2. Objective and Research Approach

The present research aims to analyze the environmental impacts of the life cycle of several asphalt pavement solutions involving innovative eco-designed asphalt mixtures, which have been previously analyzed for their mechanical properties, in the binder and base layers, and find out which solution shows the lowest environmental impacts and the highest service life. To overcome the aforementioned issues, the life cycle impacts of the pavement solutions were assessed under the same boundary conditions, in the same geographic area, and considering the same sources of primary data and the actual sites/facilities from which the waste was supplied.

**Figure 1:**
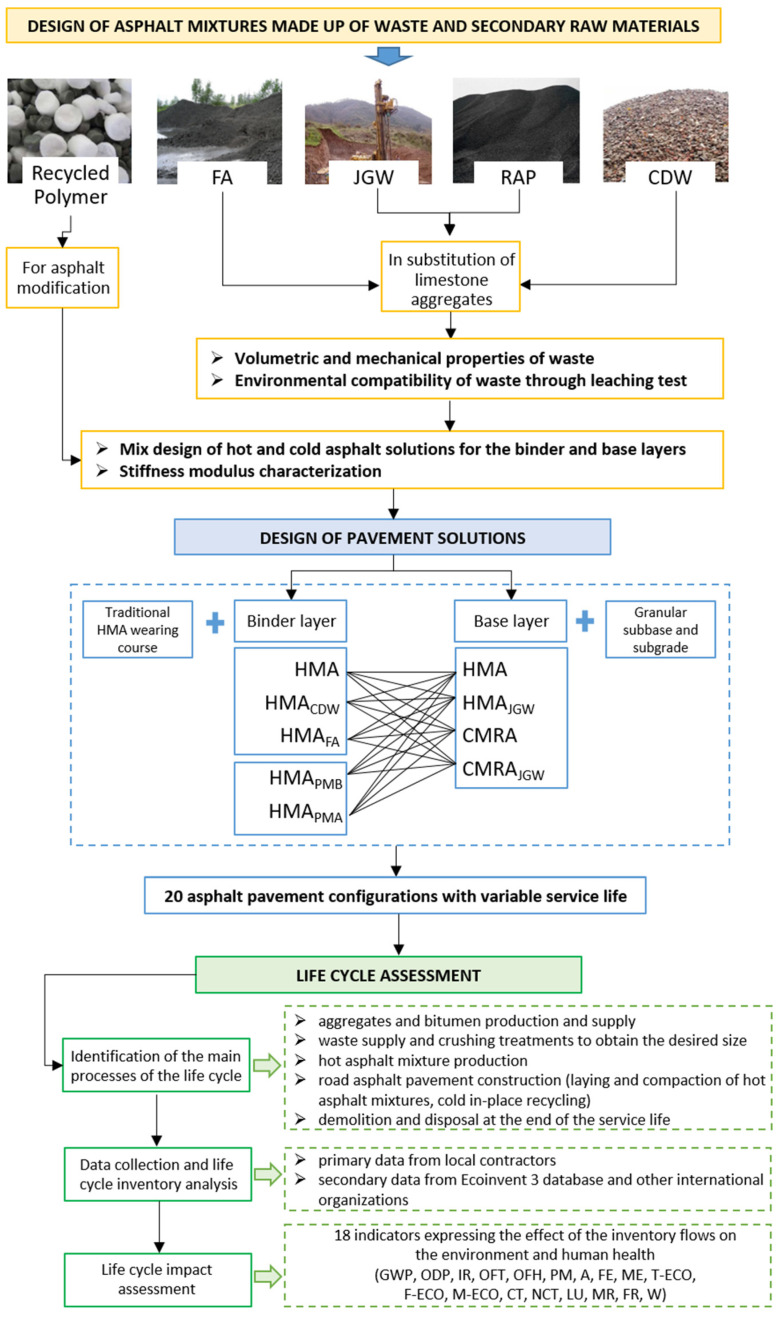
Workflow of the research.

The research study consisted of three main steps (see the workflow diagram presented in Figure 1), which were based on the circular economy objectives formulated by the European Union for reducing the consumption of resources and the impact of waste on the environment for the road asphalt pavements maintenance.

- Step 1. A total of four marginal materials were investigated and reused in substitution of natural aggregates and fillers: CDW (the waste produced during the demolition of concrete structures), FA (the residue of coal combustion in thermoelectric power plants), RAP, and JGW (the waste produced during jetting operations for soil consolidation). In addition, commercially produced recycled plastic pellets were adopted for the modification of hot asphalt mixtures. Subsequently, nine asphalt mixtures were designed through laboratory studies: Five for the binder layer (HMA, the traditional asphalt mixture, two hot mix asphalts made up of recycled aggregates, HMA_CDW_ HMA_FA_, and two modified hot mix asphalts, HMA_PMB_ and HMA_PMA_, respectively manufactured through wet and dry modification processes) and four for the base layer (HMA; HMA_JGW_, CMRA, and CMRA_JGW_). The stiffness modulus of the designed road asphalt mixtures was then assessed through an indirect tensile test (EN 12697-26—Annex C).
- Step 2. The above-mentioned asphalt mixtures were adopted for the design of asphalt pavement solutions assuming the linear elastic multilayer deriving from Boussinesq theory as the structural model of the pavement, where each layer is characterized by a seasonal stiffness modulus and Poisson’s ratio, for the calculation of the stress–strain state to predict the service life in compliance with the fatigue damage and rutting accumulation laws.
- Step 3. LCA methodology was applied to a one-kilometer road section paved with the designed asphalt solutions. Inventory flows were calculated for all phases of the life cycle, starting with the production of virgin materials (natural aggregates and binders), the supply and treatments of the mentioned waste (CDW, RAP, JGW, and FA), the production of recycled polymer pellets, until the production and laying of asphalt mixtures and disposal of the road pavement at the end of the service life. An impact assessment was performed according to ReCiPe methodology, which converted the inventory flows (emissions of hazardous substances, consumption of natural resources, and waste production) into 18 impact category indicators.

## 3. Materials and Methods

### 3.1. Natural and Recycled Aggregates

In the present work, several asphalt mixtures were designed using waste in partial substitution of natural ones, aiming, on the one hand, to lower the consumption of non-renewable natural resources and on the other hand to reduce the emission of pollutants and the consequent effects on the environment. The natural aggregates adopted in the study were limestone aggregates extracted from a quarry near Caserta in Southern Italy, whose physical and mechanical properties are reported in Table 1.

Four wastes available locally (either supplied from external sources or produced directly in the construction site under analysis) were selected and investigated for subsequent reuse, as follows:

- RAP was milled from the existing deteriorated asphalt pavement’s wearing and binder layers and reused as a recycled coarse aggregate directly in the same construction site without any additional crushing actions. In particular, given the high Los Angeles value (see Table 1), namely the aggregate toughness and abrasion resistance, it was used as a coarse aggregate substitute in the base layer, which is less affected by traffic wearing actions.
- CDW was supplied to the asphalt plant from a distance of 20 km and then milled until an aggregate distribution was reached that entirely passed at a 0.063 mm sieve size. Looking at the results presented in Table 1, the CDW filler has a sand equivalent value very similar to the limestone’s; in addition, the Rigden voids value of the CDW suggests higher optimum bitumen content and higher stiffness of the optimized mixture. Therefore, it was selected to substitute limestone filler in the binder layer.
- FA resulted from a thermoelectric power plant after a coal combustion process and was supplied to the asphalt plant located 100 km away; its particle size range was between 10 and 100 µm, therefore it was reused as a substitute for natural fillers without additional size reduction actions. The highest sand equivalent value (see Table 1) indicates the absence of almost any organic material, which complies with the nature of combusted particles and makes FA a high-quality fine aggregate to be used in the binder layer.
- JGW is initially produced as a spoil of water, soil, and cement after high-pressure injection for ground consolidation works; once it dries out, it is either supplied to the asphalt plant (that is 20 km away from the JGW production site) or cold-mixed with RAP directly onsite and milled for 2 h until the filler size is obtained. The physical properties shown in Table 1, in particular the higher Rigden voids value than that of the limestone filler, suggest its potential to enhance the stiffness of the mixture.

The four wastes also underwent an environmental compatibility check before their reuse through leaching test analysis (EN 12457-2), which returned the concentrations of several pollutants, such as heavy metals and organic compounds, into water. All the above-mentioned waste produced leaching concentrations systematically lower than the quantification limits of the instruments; the substances that were found in relevant concentrations in the eluate, but still lower than the limits established by the Italian Ministerial Decree issued on 5 February 1998, are shown in Table 2.

### 3.2. Binders

A 50/70 penetration-grade neat bitumen and a hard-modified bitumen with SBS virgin polymer were adopted as a natural binder for hot asphalt mixtures, respectively, as a benchmark for non-modified and polymer-modified asphalt mixtures; cationic bitumen emulsion and Portland cement 325 R were used as main binders into cold mixtures. The main properties of the binders are shown in Table 3.

### 3.3. Recycled Plastic Pellets for Asphalt Mixture Modification

The polymer compound used in this study for asphalt dry modification was composed mainly of: (a) Polyethylene terephthalate (PET) recycled plastics subjected to a crushing treatment process to achieve final dimensions lower than 2 mm; (b) a mixture of optimized plastomeric polymers and copolymers such as Low-Density Polyethylene (LDPE) and EVA; and (c) additives of different types to ensure that the final polymer compound would be in the form of semi-smooth and flexible granules. The main properties in terms of melting point and apparent density are displayed in Table 3.

### 3.4. Design of Asphalt Mixtures

The first step of the mix design procedure consists of defining an aggregate size distribution, aiming to reuse as much waste as possible in compliance with the specifications of UNI EN 13108-1 for the pavement layer for which the asphalt mixture is designed.

The optimum bitumen content (OBC) of hot asphalt mixtures for the binder layer (HMA, HMA_CDW_, HMA_FA_, HMA_PMB_, and HMA_PMA_) and base layer (HMA and HMA_JGW_) corresponded to the maximum Marshall Stability value (EN 12697-34) (see Table 4a,b).

Instead, the procedure adopted for the definition of the optimum cement, water, and bitumen emulsion content of the cold asphalt mixtures for the base layer (CMRA and CMRA_JGW_) is briefly reported as follows:

- Asphalt specimens are compacted using giratory compaction at 180 revolutions with cement content in the range of 0.5–1.5 wt.% and water content in the range of 4–6 wt.%
- Optimum cement and water content are selected in correspondence with maximum specific gravity.
- Asphalt specimens at the optimum cement and water content are compacted using giratory compaction at 180 revolutions with bitumen emulsion content in the range of 3–6 wt.%
- Optimum bitumen emulsion content is in correspondence with the closest specific gravity to that of the reference HMA (2.50 g/cm^3^).

The aggregate size distribution and the mix design results are shown in Table 4a for all the hot asphalt mixtures for the binder layer and in Table 4b for the hot and cold asphalt mixtures for the base layer.

### 3.5. Stiffness Modulus Characterization

Asphalt pavement distresses are strictly linked to the ability of the pavement layers to spread the load applied by the vehicle’s axles passages and to control the stress–strain state induced in the asphalt materials. One of the mechanical tests that best represents the dynamic response of asphalt materials under repeated loading is the Indirect Tensile Stiffness Modulus (ITSM) test performed according to EN 12697-26—Annex C. The test consists of the application of indirect tension to asphalt specimens (a cylindrical shape with a diameter of 150 mm and a height equal to 63 mm ± 1 mm) The ITSM was determined as the average ITSM calculated at each load pulse by using Equation (1).

(1) $$ITSM=\frac{F\cdot \left(\nu +0.27\right)}{z\;\cdot h}\;\left(MPa\right)$$

where:

*F* is the peak value of the applied vertical load (N);

*z* is the amplitude of the horizontal deformation obtained during the load cycle (mm);

*h* is the mean thickness of the specimen (mm);

*ν* is the Poisson’s ratio, set equal to 0.35 according to UNI EN 12697-26.

The test was performed at four temperatures (5, 10, 20, and 30 °C), which represent the average temperature conditions of the pavement in Southern Italy across the four seasons (winter, spring, autumn, and summer, respectively).

The results of the stiffness modulus characterization are reported in Table 5 for all the asphalt mixtures under exam and were of fundamental importance during the subsequent design of asphalt pavement solutions to address the linear elastic constitutive law of each asphalt material.

### 3.6. Design of Pavement Solutions

Road pavement design relies on the definition of a structural model, where each material is characterized by its stiffness modulus (ITSM) and Poisson’s ratio, to define the stress–strain state in the pavement under the equivalent standard axle load (ESAL) and the thickness required by each pavement layer to contain the tensile stresses at the bottom of the base layer, which leads to fatigue cracking, and the compression stresses at the top of the asphalt layers, responsible for the rutting phenomenon.

In the present work, the designed asphalt mixtures are used in the binder and base layer of asphalt pavements, while the wearing course is made up of traditional HMA, whose mix design and mechanical properties were assessed in Russo et al. [36] and Veropalumbo et al. [13]. Moreover, the asphalt pavement is laid on an existing granular subbase with a Young elastic modulus of 116 MPa, and a subgrade with a deformation modulus of 50 MPa.

In detail, the m asphalt mixtures for the binder layer (with m equal to 5) and the n asphalt mixtures for the base layer (with n equal to 4) are combined into mxn solutions, that is, 20 pavement stratigraphies.

The cumulative fatigue damage (*CD*), which must be kept below 1 to avoid excessive deterioration of the surface quality, is determined as the sum of the seasonal Di, namely the share of relative damage produced by the ESALs passings during the *i*-th season, according to the Miner law, which is reported in Equation (2).

(2) $$CD=\overset{S}{\underset{i=1}{\sum }}\frac{n_{i}}{N_{i}}$$

where:

*n_i_* is the number of ESAL passages in the *i*-th season, which is computed through the AASHTO Design method [37] supposing that the ESAL passings are evenly distributed between the four seasons;

*N_i_* is the number of ESAL passages that leads to an extension of fatigue cracking damage up to 10% of the lane area of the road pavement, determined according to the Asphalt Institute fatigue prediction law [38] through the average ITSM and the tensile strain at the bottom of the base layer resulting from the linear elastic multilayer model.

The rut depth (*RD*) at the end of the service life, which should never be deeper than 2 cm, is predicted through the Vaerstaten law, as shown in Equation (3).

(3) $$RD=\underset{j}{\sum }\underset{i}{\sum }\varepsilon_{ij}\cdot h_{j}$$

where:

*ε_ij_* is the permanent deformation in the *i*-th season that is accumulated in the *j*-th asphalt layer of the pavement after the mentioned ESAL passings, determined according to the Kaloush and Witczak model [39] based on the vertical compressive strain obtained from the linear elastic multilayer model;

*h_j_* is the thickness of the *j*-th asphalt layer.

The pavement design results are reported in Table 6 in terms of thicknesses of the asphalt layer, the mean and standard deviation of the service life, CD, and RD. It is worth mentioning that the combination of HMA_PMB_ binder layer with cold recycled mixtures for the base layer (CMRA and CMRA_JGW_) posed some questions about the structural compatibility of these materials since the application of the ESAL induced a tensile stress state in the binder layer that was not compatible with the structural integrity of the pavement in the long term. Therefore, two alternative pavement stratigraphies were obtained for the combination of HMA_PMB_ and CMRA/CMRA_JGW_ (see Table 6).

## 4. Life Cycle Assessment

The LCA methodology was applied to quantify the environmental impacts of the production, construction, demolition, and disposal stages of the designed pavement solutions. According to the standardized framework of EN 14040, LCA involves three mandatory phases: (i) The goal and scope definition phase, which sets the motivation of the analysis, quantifies the reference unit, and defines the system boundary; (ii) the life cycle inventory (LCI), which involves the data collection and the definition of the flows of materials (mainly input flows of minerals and fossil fuels) and substances (outputs emitted to air, water, and soil) that stream through the unit processes of the system; and (iii) the life cycle impact assessment (LCIA), which entails the selection of impact categories, category indicators, and characterization models, the assignment of LCI results to the selected impact categories (classification), and the calculation of category indicator results (characterization) to convert the input and output flows of the inventory into understandable environmental impact indicators.

### 4.1. Goal and Scope Definition Phase

First, as mentioned in Section 2, the motivation behind the study was to compare, under homogeneous hypotheses and system conditions, different sustainable asphalt solutions involving the use of waste and recycled modification polymers combined in the binder and base layers of road pavements and find out which pavement configurations has the lower environmental impacts in terms of conservation of the natural environment and resources.

**Figure 2:**
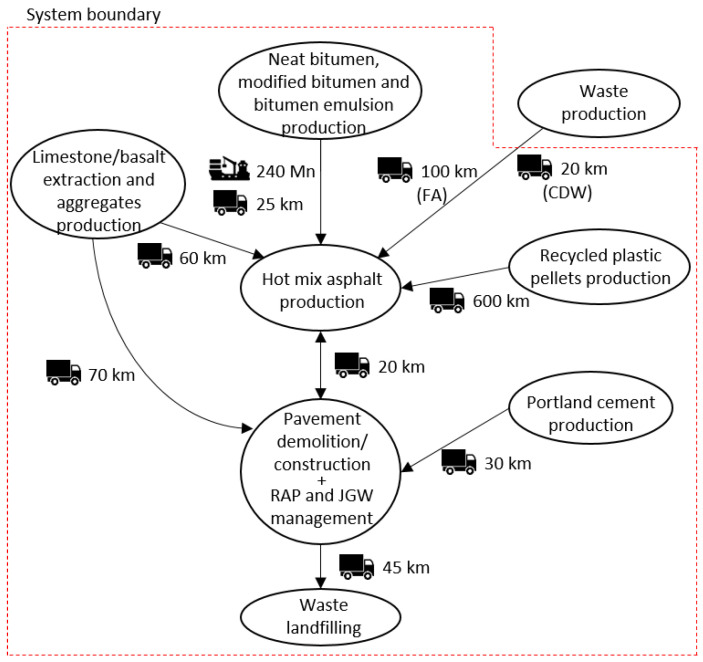
Overview of the facilities involved in the system boundary (considering all the possible phases of the life cycle) and relative transportation distances of the case study.

In order to do so, the 20 pavement configurations were assumed as alternative maintenance solutions for the same case study, which involved the demolition and reconstruction of the wearing course, binder, and base layers of an existing pavement on a 1-km section of a single-carriageway road (width equal to 10.5 m) located in southern Italy. The main processes included in the system boundary and the relative distances between the local facilities involved in the analysis are reported in Figure 2.

### 4.2. System Description and Data Collection

The LCI phase involves the collection of primary and secondary data for the modelling of the flows that stream trough the unit processes of the system, namely input flows, such as materials and fossil fuels (crude oil, natural gas, and coal), and output flows, such as waste and pollutant emissions to air (CO_2_, CO, CH_4_, NO_x_, SO_2_, NH_3_, polycyclic aromatic hydrocarbons (PAH), non-methane volative organic compounds (NMVOC), and particulate matter) and water (chemical oxygen demand (COD), biological oxygen demand (BOD5), and nitrogen and sulfur compounds).

The following subsections give a description of the unit processes involved in each phase of the life cycle and the relative data sources used to compile the LCI.

For the deepening of the complete list of data sources, refer to Table S1 in the Supplementary Material. Note that each manufacturing phase also includes the flows associated with the transportation to the next facility/construction site.

#### 4.2.1. Natural Aggregates Production

This phase involves the operations of rock mining, transportation of raw rocks to the aggregates production plant, the crushing and sieving phase, the loading of the aggregates into the trucks, and the transportation of aggregates either to the asphalt plant, for hot asphalt production, or to the construction site, for the granulometric correction of RAP’s aggregate size distribution in cold in-place recycling operations. In particular, two different types of coarse aggregates were considered: Limestone aggregates, included in the mix design of the binder and base layer asphalt mixtures, and basalt aggregates, which are usually used in the wearing course due to high abrasion and crushing resistance. Instead, natural sand and filler always had a calcareous origin. The inventory data for the production on 1 kg of coarse limestone and basaltic aggregates were gathered from the Ecoinvent 3 database [40] and referred to the amount of aggregates in each asphalt mixture (see Table 4a,b).

#### 4.2.2. Management and Supply of CDW, JGW, FA and RAP

As already mentioned in Section 3.1, the CDW and FA were supplied from external facilities/sites, so the inventory flows associated with their treatments and transportation phases were computed only when the analyzed pavement configuration involved the reuse of these secondary materials in the binder layer (HMA_CDW_ and HMA_FA_).

On the other hand, the RAP and the JGW originated from the construction site under analysis (7098 and 210 t, respectively), therefore their management operations either corresponded to disposal (for the pavement configurations with HMA base layer) or partial/total recycling (for HMA_JGW_, CMRA and CMRA_JGW_ base layers).

All the inventory flows of the crushing (mobile diesel-powered jaw mill with a productivity of 150 t/h) and transportation phases (trucks with capacity of 20 t travelling the distances reported in Figure 2) were selected from the Ecoinvent 3 database and referred to the amount of waste reused in substitution of natural aggregates (see Table 4a,b).

#### 4.2.3. Bituminous Binders Production and Supply

The production of petroleum derivatives takes place within the refinery, where the extracted crude oil is distilled into several products: The heaviest fraction is the neat bitumen. In the present analysis, the neat bitumen is used to bind the aggregates of several hot asphalt mixtures for the wearing course (HMA) binder (HMA, HMA_CDW_, HMA_FA_, and HMA_PMA_) and base layer (HMA and HMA_JGW_) that make up the corresponding asphalt pavement configurations. Instead, HMA_PMB_ and the cold mixtures for the base layer (CMRA and CMRA_JGW_) required SBS-modified bitumen and bitumen emulsion, respectively. Both the modified bitumen and the bitumen emulsion were obtained from the high shear milling of the neat bitumen with SBS-polymers or hot water, respectively. After that, the binders were hauled to the asphalt plant (see Figure 2). The inventory data for the production on 1 kg of neat bitumen, modified bitumen, and bitumen emulsion were gathered from the European Bitumen Association [41] and referred to the binder content of each asphalt mixture (see Table 4a,b).

#### 4.2.4. Cement Production and Supply

The manufacturing of Portland cement involves the burning of a mixture of crushed rocks in a kiln, and the grinding of the burned product, known as clinker, together with a small percentage of gypsum. The cold mixtures of the base layer (CMRA and CMRA_JGW_) required Portland cement (according to the percentage reported in Table 4b) to speed up the breakage of bitumen emulsion and to strengthen the final mixture. For that reason, Portland cement was hauled directly to the construction site for cold-in place recycling operations. The inventory data for the production of 1 kg of Portland cement were found in the Ecoinvent 3 database.

#### 4.2.5. Recycled Polymer Pellets Production

The conversion of waste plastic into recycled pellets for asphalt dry modification (HMA_PMA_) mainly involves the processes of waste collection, sorting, shredding, and pelletization (in which plastic shreds are heated up to 190 °C and extruded to become pellets). In the present work, the inventory data were gathered both from the Ecoinvent 3 database (for the collection, sorting, and shredding of waste plastics) and from Santos et al. [42] for the electricity and water consumption and the waste generation during the pelletization process. The amount of polymer pellets that make up HMA_PMA_ is reported in Table 4a.

#### 4.2.6. Hot Mix Asphalt Production

All the hot asphalt mixtures for the wearing course (HMA), binder (HMA, HMA_CDW_, HMA_FA_, HMA_PMB_, and HMA_PMA_), and base layer (HMA and HMA_JGW_) were produced in a batch plant at a high temperature (160–180 °C). Some of the useful data used to quantify the inventory flows were surveyed at a local asphalt plant: (a) The productivity of a wheel loader that handles the aggregates from the stockpiles to the plant supply system, equal to 60 m^3^/h and powered by diesel fuel, (b) the natural gas consumption, equal to 8.79 m^3^ for each tonne of asphalt mixture, required for drying the aggregates in the drum dryer, and (c) the electricity consumption of the asphalt plant, equal to 4.37 kWh/t of asphalt mixture, that powers the high-temperature storage and feeding system of bitumen and the mixing of aggregates, filler, and bitumen for the production of the hot asphalt mixture. All the input and output inventory flows were then selected for the corresponding operations from the Ecoinvent 3 database. In addition, the emissions to air of 16 mg/kg of NMVOC, 40 mg/kg of PM10, and 2 mg/kg of PM2.5 were considered during hot asphalt mixture production, as reported in tier 2 emission factors by the United States Environmental Protection Agency (2004) for the batch hot asphalt mixing plant setup with a pollutant abatement system. The amount of hot asphalt mixtures produced at the asphalt plant was estimated based on the thickness of each layer (see Table 6).

#### 4.2.7. Pavement Construction

Road pavement construction operations differ for hot in-plant produced and cold in-place recycled asphalt mixtures in terms of the machinery involved for the laying and compaction to reach the desired density.

Looking at the construction operations with hot asphalt, the paving and the roller machines work in series to place the asphalt on the surface and compact it. The survey of actual paving operations allowed estimating the productivity of the whole construction for each pavement layer (351 t/h for the wearing course, 205 t/h for the binder, and 117 t/h for the base layer). The type and power and the machinery helped in selecting the list of unit inventory flows from the Ecoinvent 3 database.

The CMRA and CMRA_JGW_ base layers, instead, are mixed and laid during cold in-place recycling operations. Cold in-place recycling involves: (i) The operation of a diesel-powered motor grader, which places the milled RAP back on the subbase surface with a productivity of 1815 m^2^/h of road surface; (ii) the mixing of RAP (and eventually JGW), natural aggregates, cement, water, and bitumen emulsion (each one of them supplied by trucks and tankers to the construction site) by a diesel-powered pulvimixer, which works 750 m^2^/h of pavement surface; and (iii) the compaction of the placed cold mixture up to the desired density reached during the mix design, involving two large pneumatic tire rollers and a large vibratory steel wheel roller, all of them with a productivity of 141 t/h.

#### 4.2.8. Demolition and Disposal to Landfill

Lastly, each asphalt pavement stratigraphy, once reaching the end of the service life (reported in Table 6), is demolished (through a milling machine with a productivity of 150 t/h), hauled to the nearest disposal site, and landfilled. As a matter of fact, the recyclability potential of the analyzed asphalt mixtures made up of waste and secondary raw materials should be further assessed. The inventory flows arising from asphalt waste landfilling were estimated from the specific section of the Ecoinvent 3 database.

#### 4.2.9. Transportation Phases

The transportation of the raw materials, semi-finished products, and waste, according to the scheme reported in Figure 2, was carried out by road through heavy vehicles, specifically dump and tank trucks, whose emissions and resources consumption were estimated from the Ecoinvent 3 database. Additionally, the bituminous binders were also transported by sea into a freighter.

### 4.3. Life Cycle Impact Assessment

The LCIA phase aims to convert the input and output flows of the LCI into impact category indicators, which are understandable measures of specific environmental problems that can affect the environment, human health, and the availability of natural resources. In this study, the impact assessment of the designed pavement solutions was performed through SimaPro 9^®^ software. Among the impact assessment models available in the literature, the Egalitarian ReCiPe [43] impact assessment method was selected both for its diffusion in the construction sector [44,45] and the wide range of environmental problems addressed by its impact categories. In ReCiPe, there are indicators at two levels: 18 midpoint indicators and 3 endpoint indicators. Midpoint and endpoint level indicators refer to two different stages in the cause–effect chain that starts from the input and output flows of the processes of the life cycle, which are converted into midpoint effects on specific environmental topics and, at the end of the cause–effect chain, produce effects on broader endpoint impact categories, such as human health, ecosystems, and resource availability. The following midpoint impact categories were considered: Climate change (GWP, kg CO_2_ eq), stratospheric ozone depletion (ODP, kg CFC11 eq), ionizing radiation (IR, kBq Co-60 eq), damage of ozone formation on terrestrial ecosystems (OFT, kg NO_x_ eq) and human health (OFH, kg NO_x_ eq), fine particulate matter formation (PM, kg PM2.5 eq), terrestrial acidification (A, kg SO_2_ eq), freshwater eutrophication (FE, kg P eq), marine eutrophication (ME, kg N eq), terrestrial, freshwater and marine ecotoxicity (T-ECO, F-ECO and M-ECO, kg 1,4-DCB eq), human carcinogenic (CT, kg 1,4-DCB eq) and non-carcinogenic toxicity (NCT, kg 1,4-DCB eq), land use (LU, m^2^ a crop eq), mineral resource scarcity (MR, kg Cu eq), fossil resource scarcity (FR, kg oil eq), and water consumption (W, m^3^).

## 5. Results

**Figure 3:**
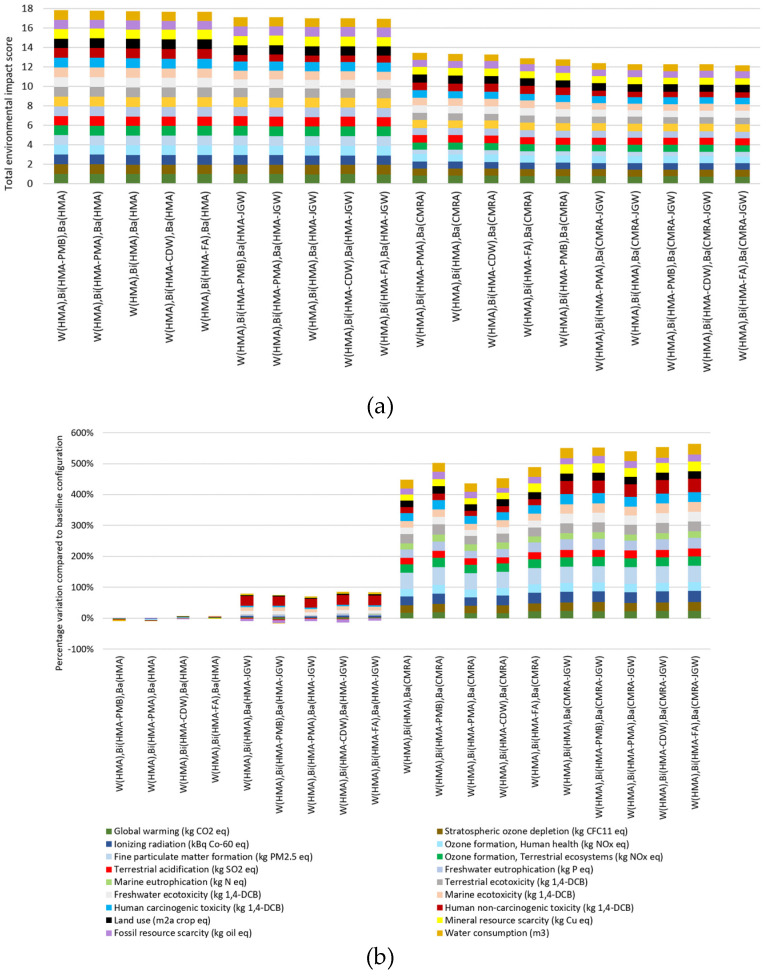
(**a**) Total environmental impact score, calculated as the sum of the normalized impact category indicators, for each pavement configuration and (**b**) percentage variations of each impact category indicator of the pavement configurations compared to the baseline pavement configuration in traditional HMA.

The overall results in terms of midpoint indicators of the comparative LCIA conducted on the twenty asphalt pavement configurations are presented in Table S2 in the Supplementary Material along with the percentage variations compared to the baseline pavement configuration involving a traditional HMA in the wearing course, binder, and base layer; the total environmental impact score IS_tot,j_ for each j-th pavement configuration, calculated according to Equation (4), is reported in Figure 3a for the 20 pavement configurations under analysis.

(4) $$IS_{tot,\;\;j}=\overset{18}{\underset{i=1}{\sum }}\frac{IC_{i,j}}{\max_{j}\left(IC_{i}\right)}$$

where:

*IC_i,j_* is the *i*-th environmental impact indicator of the *j*-th pavement configuration, with *i* in the range [1;18] and j in the range [1;20];

*max_j_(IC_i_)* is the maximum value of the *i*-th environmental impact indicator between the *j* pavement alternatives.

The lower the *IS_tot,j_*, the better the overall environmental performance.

Additionally, Figure 3b shows the percentage variations of all the impact category indicators of each pavement configuration compared to the baseline pavement configuration made up of traditional HMA.

Looking at Figure 3a,b, it is possible to observe that all the pavement configurations in which waste are reused in place of natural aggregates into asphalt mixtures for the binder (HMA_CDW_ and HMA_FA_) and base layer (HMA_JGW_, CMRA, and CMRA_JGW_) lower all the impact category indicators (from −0.5% up to −32%, −18% on average) compared to the asphalt pavement made up entirely of traditional HMA.

In particular, focusing on the alternative binder layer asphalt mixtures, the HMA_CDW_ gave smaller benefits, showing an average reduction of all impact category indicators equal to 0.5% with respect to those of the HMA binder layer, while HMA_FA_ lowered the indicators by 1.2% on average, again compared to those of HMA.

In particular, the greatest benefits of HMA_CDW_ are in terms of W, FE, ODP, and CT, which are respectively lower by 2.1, 1, 0.6, and 0.5% compared to those of the HMA binder. In detail, the 4% reduction of the natural limestone filler in the asphalt mixture affects the consumption of water during rocks crushing (−3.5%), the output emissions to air of PAH and chlorofluorocarbons (−13.5%), and the emissions into the water of phosphorous compounds (−1.5%) compared to those of the HMA binder.

The HMA_FA_ adds more benefits to the environmental impact in terms of lower OBC (−0.25% compared to that of HMA binder), which mainly affects FR and GWP, 1.4 and 1.8% lower, respectively, compared to those of the HMA binder and a further 2.9 and 0.3% lower than HMA_CDW_.

Instead, the wet (HMA_PMB_) and dry (HMA_PMA_) modification of asphalt mixtures entails additional environmental burdens compared to the traditional HMA. In addition, the pavement configurations using recycled plastic pellets in the binder layer (combined with HMA, HMA_JGW_, CMRA, or CMRA_JGW_ base layers) do not show a significant improvement of the impact category indicators compared to those with the HMA_PMB_ binder (−0.2%), above all due to high environmental burdens of the plastic pelletization process, long transportation distance (see Figure 2), and reduced thickness of the pavement configurations with HMA_PMB_ binder and cold-recycled base layers (see Table 6). Moreover, the avoided amount of plastic waste going to landfill is not included in the analyzed system boundary, therefore some of the positive benefits of using recycled plastic pellets for asphalt dry modification instead of bitumen modification are not accounted for in the present analysis.

As clearly displayed in Figure 3a,b, the main source of variability of the overall environmental impact was the adoption of the cold in-place recycling technology for the construction of the base layer, which lowered on average all the impact category indicators: −26% for CMRA versus HMA base layer, −31% and −28% for CMRA_JGW_ versus HMA and HMA_JGW_ base layer, respectively.

**Figure 4:**
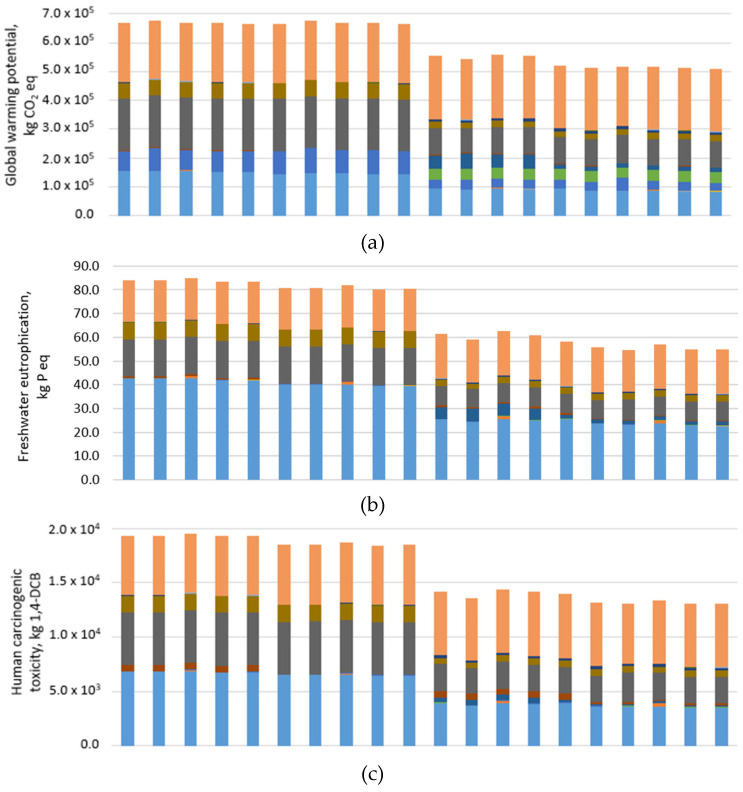

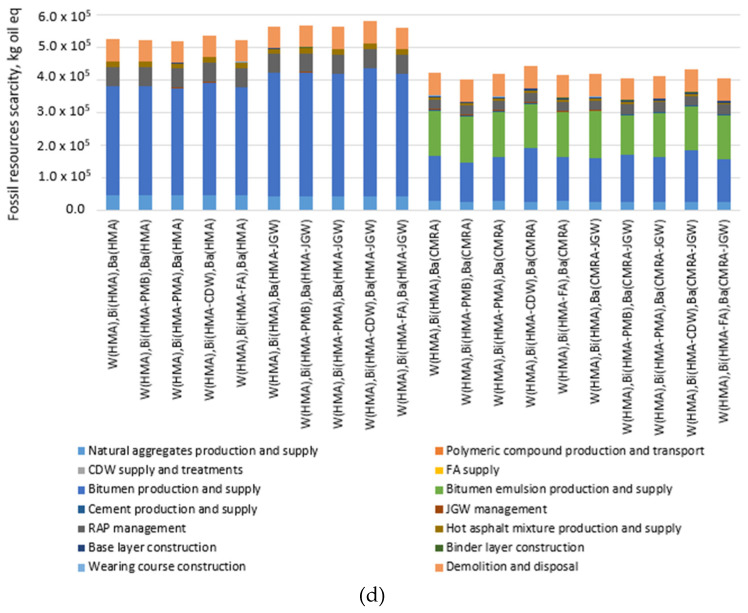
Process contribution for each pavement configuration to (**a**) global warming potential, (**b**) freshwater eutrophication, (**c**) human carcinogenic toxicity, and (**d**) fossil resources scarcity.

The impact category indicators that show that the maximum variations between the alternative pavement configurations are the GWP, which represents the heat absorbed by any greenhouse gas in the atmosphere, TECO, which is the effect of several chemical substances to terrestrial organisms and plants, CT, which stands for the effect of toxic chemicals that are linked to increased cancer occurrence, and FR, which is the effect of anthropogenic activities on the depletion of fossil resources. The GWP, TECO, CT, and FR have been reported for each process of the life cycle in Figure 4a–d, respectively. The following considerations can be drawn:

- The values of GWP, TECO, and CT are linked respectively to the emissions to air of greenhouse gases (CO_2_, CH_4_, N_2_O), the emissions to the soil of nitrogen and phosphorous compounds, and the emissions to air of PAH and particulate matter. The sum of the inventory flows of aggregates’ production and supply and asphalt waste landfilling (RAP management and disposal of the asphalt pavement at the end of the service life) made up 77%, 88, and 89% of the total GWP, TECO, and CT, respectively (averaged on all the pavement configurations). On the contrary, the FR indicator was mainly affected by the bitumen production and transportation process (65% of total FR for the pavement configurations with HMA and HMA_JGW_ base layer, 33% of total FR for those with CMRA and CMRA_JGW_ base layer) since both the energy resources and the raw material have fossil origin.
- Comparing the pavement configurations made up with hot asphalt mixtures only, the GWP (see Figure 4a) shows the lowest variation between the alternatives; in particular, the solution that minimizes the GWP is the one that combines the HMA_FA_ binder with the HMA_JGW_ base layer, which saves around 10 t CO_2_ eq in the phase of aggregates’ production and supply (6% lower amount of natural filler), 8.3 t CO_2_ eq during the bitumen production and supply (0.25% lower OBC), and 2.9 t CO_2_ eq during the JGW management phase (around 200 t of JGW are reused in the HMA_JGW_ base layer) compared to the traditional HMA configuration (−0.5% globally on the life cycle). Looking at the corresponding inventory flows, the best performance of the HMA_FA_-HMA_JGW_ pavement configuration is achieved in terms of CH_4_ emissions during natural filler production, passing from 320 kg for the traditional HMA pavement to 295 kg (−8% compared to those of the traditional HMA pavement).

The complete dataset showing the contribution of each process of the life cycle to all impact category indicators for all pavement configurations is reported in the Supplementary Materials from Tables S3 to S22.

A separate discussion can be carried out comparing the asphalt pavement configurations with the cold base layers to those with the hot asphalt base layers. Looking at the GWP indicator, the impact of the production and supply of raw materials (aggregates, bitumen, bitumen emulsion, and cement) for the pavement configurations with a CMRA base layer is almost the same than that of the HMA and HMA_JGW_ base layers (−6.3% on average), despite the lower amount of natural aggregates (−59 t CO_2_ emitted to air compared to HMA base layer) and the use of bitumen emulsion in the base layer (−42 t of CO_2_ emitted to air during the production of each tonne of bitumen emulsion compared to the production of 1 t of bitumen). This depends on the addition of Portland cement to the CMRA, whose production and supply accounts for 9% of the total GWP of the life cycle despite the very low mass percentage in the mixture (see Table 4b). Nevertheless, the total GWP of the solutions with the CMRA base layer is, on average, 18% lower than that of the HMA and HMA_JGW_ base layer, above all due to the avoided landfilling of 3549 t of RAP, which is reused to make up the cold recycled base layer.

Comparing the pavement configuration with the CMRA_JGW_ to those with the CMRA base layer, two main phases of the life cycle are responsible for the further 7.2% average reduction of GWP: Aggregates’ production (due to the substitution of natural filler with JGW filler, see Table 4b), which entails an 11.5% reduction of CH_4_ emissions, and cement production, whose content is lowered from 1.5% to 0.5% by the weight of aggregates (see Table 4b) and cuts the NO_x_ emissions by about 52 kg (67% reduction) compared to the CMRA base layer.

Looking at the FE indicator (Figure 4b), a more marked variation can be seen for all the pavement configurations with the HMA_JGW_ base layer compared to those with the HMA base layer (−4% on average). That depends, firstly, on the lower amount of landfilled JGW, which entails the reduction of 2.2 kg of phosphorous compounds emitted into the water when JGW is partially recycled into the HMA_JGW_ base layer compared to the HMA base layer, causing an average reduction equal to 0.7 kg P eq for each functional unit.

The FE reduction also depends on the lower emissions during aggregates’ production; in particular, the solutions with the HMA_JGW_ base layer reduce, on average, the amount of nitrogen and phosporous compounds emitted in water by 20 and 5.7%, respectively, saving around 2.5 kg P eq. The further reduction of the natural filler in the binder layer when using HMA_CDW_ or HMA_FA_ corresponds to an additional 3 kg of phosphorous compounds not emitted in water, which makes them the preferable pavement configurations to minimize freshwater eutrophication.

Looking at the comparison between the solutions with the cold- and hot-produced base layers in terms of the FE indicator, Figure 4b highlights an average 30% reduction for all the pavement configurations with the cold base layers compared to those with HMA and HMA_JGW_ base layers. In particular, the substitution of natural aggregates with RAP lowers the emissions in the water of specific substances in all the pavement configurations with the CMRA (on average −7% and −44% nitrogen and phosphorous compounds, respectively, compared to those with HMA base layer) and CMRA_JGW_ base layer (on average −20% and −41% nitrogen and phosphorous compounds, respectively, compared to the HMAJ base layer, and −21% and −41% of the same substances compared to HMA_JGW_ base layer). Between the pavement configurations with the CMRA_JGW_ base layer, the one with the HMA_PMB_ binder shows the greatest benefits on the FE indicator, 2 and 25% lower, respectively, compared to that of the HMA-CMRA_JGW_ pavement configuration and traditional HMA stratigraphy, above all due to the lower thickness of the pavement stratigraphy achieved during the design phase (see Table 6).

A synthetic indicator of the toxicity of pollutant emissions on human health is the CT indicator; as shown in Figure 4c, the CT indicator is significantly affected by the JGW management phase (on average 4% of the whole life cycle), which accounts for 7.1 kg of the total particulate matter emitted to the air when the totality of JGW is landfilled in the case of HMA base layer; between the asphalt mixtures for the binder layer, the one that performs worse than the others in terms of CT indicator is the HMA_PMA_, in which the polymeric pellets production accounts for 8 kg of total particulates and 0.2 kg of PAH emitted to air, especially during plastic shredding, sorting and transportation of the finished product (despite the very low amount included in the asphalt mixture, see Table 4b).

Looking at the asphalt pavement configurations involving the cold-recycled base layers, the average CT reduction is equal to 27.4 and 32% for the CMRA and CMRA_JGW_, respectively compared with traditional HMA stratigraphy; it is worth mentioning that the hot asphalt base layer construction phase only accounts for 0.2% of the overall CT of the life cycle, while the cold in-place recycling operations make up 2% of the CT of the life cycle due to the extensive use of heavy machinery (grader, puvimixer, and three rollers) and lower productivity of the construction operations (see Table S1 in Supplementary Material) compared to the HMA/HMA_JGW_ base layer construction. Nevertheless, the pavement solutions that minimize the CT indicator are the ones that combine the CMRA_JGW_ base layer with either the HMA_CDW_ or the HMA_FA_, which show, on average, 4% lower particulate emissions during aggregates production and supply compared to the HMA-CMRA_JGW_ stratigraphy.

Lastly, Figure 4d shows the results in terms of the FR indicator, which describes a completely different scenario from the previous indicators. In fact, comparing the pavement configurations made up entirely of hot asphalt mixtures, it can be noted that the HMA_JGW_ base layer increases the FR by 8% on average compared to the HMA base layer, above all due to the 13.5% increase of the crude oil consumption to produce bitumen according to the OBC (see Table 4b). Instead, both the HMA_FA_ and HMA_PMA_ binder layers lower the FR indicator on average by 1% compared to the HMA binder, respectively, for the lower OBC (see Table 4a) and the slight change in the volumetric composition of the mixture (−8 t of crude oil consumption during aggregates and bitumen production) due to polymer addition.

The best performance in terms of the FR indicator is shown for the pavement configurations with the cold asphalt base layer, for which the crude oil consumption associated with the bituminous binders production (bitumen for the wearing course and a binder layer plus bitumen emulsion for the cold base layer) decreases by 21% (−76 t of crude oil) compared to the average crude oil consumption when using the HMA/HMA_JGW_ base layer. FR reductions range from −15% in the case of the HMA_CDW_-CMRA stratigraphy, up to −23% for HMA_PMB_-CMRA_JGW_ when compared to that of the traditional HMA-HMA configuration.

## 6. Discussions

**Figure 5:**
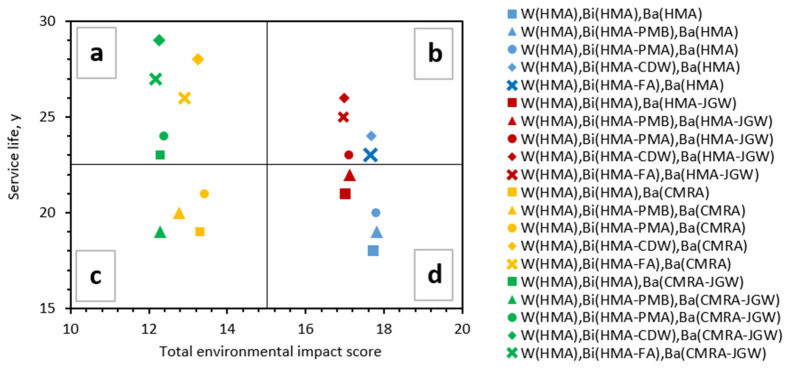
Total environmental impact score versus service life of each pavement configuration: Identification of the four regions of the plot (**a**–**d**).

The overall environmental sustainability of the pavement solutions that combine traditional and innovative eco-designed asphalt materials must be addressed jointly with the service life of each of the mentioned solutions. The plot in Figure 5 is partitioned into four regions, established based on subjective thresholds to identify four groups of alternatives: (a) Alternatives with a low environmental score (good environmental performance) and high service life; (b) alternatives with a long service life but poor environmental performance; (c) alternatives with a low environmental impact score, but short service life; and (d) alternatives that have, at the same time, the poorest environmental performance and the shortest durability.

Between the solutions falling in region “a”, the pavement configuration with HMA_CDW_ and CMRA_JGW_ as the binder and base layer, respectively, is the one that, above all, reaches the longest service life (29 years) and almost the best environmental performance (with 1% higher impact category indicators on average compared to those of the HMA_FA_ binder layer, mainly due to higher OBC and additional crushing treatments to reuse CDW as a filler).

In general, all the pavement configurations built with HMA_CDW_ or HMA_FA_ binders, combined with CMRA or CMRA_JGW_ base layers, fell into region “a”. Moreover, the reuse of the JGW in the cold recycled base layer also gave significant benefits to the service life of the pavement configurations with the HMA_PMA_ and traditional HMA binder, which are the most environmentally friendly alternatives without reusing any recycled materials in the binder layer.

Looking at region “b”, the best-compromise alternatives are the ones that combine the HMA_JGW_ base layer with the HMA_CDW_ and HMA_FA_ binders. Significantly lower service life (−2 years) and environmental benefits (+5% on average for all environmental impact indicators) are achieved when no JGW is recycled in the base layer; lastly, the use of recycled polymers into the binder layer (HMA_PMA_), combined with the HMA_JGW_ base layer, improves the service life compared to the traditional HMA (+2 years) and HMA_PMB_ (+1 year), without significantly affecting the environmental burdens.

The regions “c” and “d” highlighted in Figure 5 correspond to the lowest service life solutions (ranging from 18 to 23 years) and mainly depend on the type of hot mix asphalt used in the binder layer (HMA, HMA_PMB_ or HMA_PMA_).

Between the solutions falling in region “c”, the solution that combines the HMA_PMA_ (binder) with CMRA (base layer) shows significant improvement both in terms of service life (+3 years) and environmental performance (on average −24% of all impact category indicators) compared to the traditional HMA configuration.

Lastly, looking at the worst alternatives in region “d”, the environmental performance and service life of traditional asphalt pavement are improved when combining the HMA_PMB_ binder and the HMA_JGW_ base layer (+4 years of service life and −3.5% on average for all impact category indicators).

## 7. Conclusions

In conclusion, the synergistic effects of several innovative and traditional asphalt mixtures for binder and base layers of asphalt pavements were analyzed from both the technical and environmental points of view by designing 20 asphalt pavement stratigraphies and assessing the environmental impacts of the life cycle of these pavement configurations, applied within a real reconstruction case study of a 1-km road section located in southern Italy.

The in-depth analysis of the life cycle, the construction of a detailed life cycle inventory, and the subsequent impact assessment phase allowed drawing the following conclusions:

- CDW recycling into an HMA_CDW_ binder layer gives considerable benefits in terms of water consumption reduction during natural aggregates production, lower emissions of PAH and chlorofluorocarbons to air, as well as lower emissions of phosphorous compounds emitted to water compared to a traditional HMA binder layer. As for the HMA_FA_ binder layer, it adds even more benefits to the overall environmental impact in terms of lower OBC (−0.25% compared to that of HMA binder), which mainly affects the consumption of fossil resources and the global warming indicator;
- The wet (HMA_PMB_) and dry (HMA_PMA_) modification of asphalt mixtures entails additional environmental burdens compared to the traditional HMA binder; nevertheless, the HMA_PMA_ lowers the human carcinogenic toxicity through the reduction of particulate matter and polycyclic aromatic hydrocarbons emitted during the recycling and production of plastic pellets compared to industrial modification of bitumen with virgin polymers;
- The main source of variability of the overall environmental impact was the adoption of the cold in-place recycling technology for the construction of the base layer, which lowered all the impact category indicators on average: −26% for CMRA versus the HMA base layer, and −31% and −28% for CMRA_JGW_ versus the HMA and HMA_JGW_ base layer, respectively. In particular, the substitution of natural aggregates with RAP lowers the emissions in water in terms of nitrogen and phosphorous compounds emitted during natural aggregates’ production and supply to the asphalt plant;
- The asphalt materials that showed the best synergy between the minimization of environmental impacts and maximization of the service life of the pavement solutions were the HMA_CDW_/HMA_FA_ combined with the cold base layers CMRA/CMRA_JGW_, increasing the service life of a traditional HMA stratigraphy by 8 years for the HMA_FA_-CMRA solution, and up to 11 years for the HMA_CDW_-CMRA_JGW_ solution.

The applied methodology could be further developed by increasing the amount of primary data, especially in relation to the treatments applied for waste crushing and supply, to improve the overall quality of the analysis. Moreover, sensitivity analyses on the boundary conditions and the transportation distances (especially the ones involving the supply of waste and secondary raw materials) could be applied to remove the dependency of the results from the specific case study and verify the consistency of the results.

## Figures and Tables

**Table 1:** Physical and mechanical properties of the limestone aggregates and waste materials after size reduction.

Property	Limestone	CDW	JGW	FA	RAP
Los Angeles value (EN 1097-2)	16	–	–	–	24
Rigden voids (EN 1097-4)	51.4	55	53	48.1	–
Sand equivalent (EN 933-8)	80	76	60	96	71

**Table 2:** Metals and organics concentrations above the quantification limits and pH of the eluate of the leaching test for the analyzed waste materials and limits set by M.D. issued on 5 February 1998.

Parameter	Unit	CDW	JGW	FA	RAP	Limits of M.D. 5 February 1998
Zinc	mg/L	0.37	0.52	0.86	<0.01	3
Chloride	mg/L	65.78	53.17	77.36	16.30	100
Nitrate	mg/L	0.10	0.09	0.15	0.05	50
Fluoride	mg/L	<0.01	<0.01	<0.01	0.52	1.5
Sulphate	mg/L	6.33	21.6	36.59	7.45	250
pH	–	5.02	10.92	6.05	6.95	12
COD ^1^	mg/L	10.3	6.8	7.99	25.5	30

^1^ Chemical Oxygen Demand.

**Table 3:** Main properties of the neat and modified bitumen, bitumen emulsion, cement, and polymeric pellets.

Property	Unit	Value	Standard
Neat bitumen			
Penetration @ 25 °C	dmm	68	UNI EN 1426
Softening point	°C	48.8	UNI EN 1427
Dynamic viscosity @ 150 °C	Pa s	0.25	UNI EN 13702
Modified bitumen			
Penetration @ 25 °C	dmm	52	UNI EN 1426
Softening point	°C	87	UNI EN 1427
Dynamic viscosity @ 150 °C	Pa s	1.38	UNI EN 13702
Bitumen emulsion			
Water content	%	40	UNI EN 1428
pH value	-	4.2	UNI EN 12850
Settling tendency at 7 days	%	5.8	UNI EN 12847
Cement			
Initial setting time	min	112	UNI EN 196-3
Compressive strength	-	-	-
at 2 days	MPa	27.8	UNI EN 196-1
at 28 days	MPa	61.2	UNI EN 196-1
Volume constancy	mm	0.52	UNI EN 196-3
Polymeric pellets			
Melting point	°C	180–190	-
Apparent density @ 25 °C	g/cm^3^	0.40–0.60	-

**Table 4:** Aggregate size distribution and mix design results for (**a**) the hot asphalt mixtures for the binder layer and (**b**) the cold asphalt mixtures for the base layer.

**(a)**						
**Mixture ID**		**HMA**	**HMA_CDW_**	**HMA_FA_**	**HMA_PMB_**	**HMA_PMA_**
Mix composition	Limestone 12/18 mm	25%	23%	23%	25%	25%
	Limestone 6/12 mm	33%	29%	29%	33%	33%
	Limestone 3/6 mm	–	13%	13%		
	Limestone sand	38%	31%	31%	38%	38%
	Limestone filler	4%	–	–	4%	4%
	FA	–	–	4%	–	–
	CDW	–	4%	–	–	–
	Bitumen wa.% *	5.00%	5.75%	4.75%	5.00%	5.00%
	Polymer pellets wb.% **	–	–	–	–	5.00%
Volumetric properties	% air voids	4.20%	5.45%	5.51%	4.20%	4.20%
	Specific gravity, g·cm^−3^	2.50	2.54	2.52	2.50	2.50
Mechanical properties	Marshall stability, daN	771.6	1245.3	989.8	1125.3	1099.1
**(b)**						
**Mixture ID**		**HMA**	**HMA_JGW_**	**CMRA**	**CMRA_JGW_**	
Mix composition	Limestone 18/31.5 mm	9%	9%	16%	16%	
	Limestone 12/18 mm	32%	32%	7%	7%	
	Limestone 6/12 mm	31%	31%	–	–	
	Limestone sand	21%	21%	–	–	
	Limestone filler	7%	–	7%		
	JGW	–	7%	-	7%	
	RAP	–	–	70%	70%	
	Bitumen wa.%	4.50%	4.85%	–	–	
	Bituminous emulsion wa.%	–	–	3.75%	5.00%	
	Cement wa.%	–	–	1.50%	0.5%	
Volumetric properties	Air voids	4.50%	5.85%	9.00%	9.00%	
	Specific gravity, g·cm^−3^	2.50	2.52	2.49	2.51	
Mechanical properties	Marshall stability, daN	750.0	864.5	902.3	956.5	

* wa.% = by the weight of aggregates; ** wb.% = by the weight of bitumen.

**Table 5:** Indirect tensile stiffness modulus results at 5, 10, 20, and 30 °C for the asphalt mixtures under analysis.

Asphalt Layer	Mixture Identification	ITSM (MPa)			
		5 °C	10 °C	20 °C	30 °C
Binder	HMA	17,000	14,832	8000	1000
	HMA_CDW_	20,647	16,500	9580	4493
	HMA_FA_	21,778	17,000	9260	3940
	HMA_PMB_	14,500	13,000	6800	2002
	HMA_PMA_	17,700	16,500	8600	1500
Base	HMA	15,500	7350	6952	2960
	HMA_JGW_	17,649	9725	8826	4204
	CMRA	5855	2970	2270	1490
	CMRA_JGW_	8684	3220	2431	1614

**Table 6:** Asphalt pavement configurations, thicknesses of the asphalt layers, mean (µ) and standard deviation (σ) of the service life, cumulative fatigue damage, and rut depth.

Asphalt Pavement Configurations ^1^	Layers’ Thickness (Cm)			Service Life (y)		CD (−)		RD (cm)	
	Wearing Course	Binder Layer	Base Layer	µ	σ	µ	σ	µ	σ
W(HMA); Bi(HMA); Ba(HMA) W(HMA); Bi(HMA_FA_); Ba(HMA) W(HMA); Bi(HMA_CDW_); Ba(HMA) W(HMA); Bi(HMA_PMB_); Ba(HMA) W(HMA); Bi(HMA_PMA_); Ba(HMA) W(HMA); Bi(HMA); Ba(HMA_JGW_) W(HMA); Bi(HMA_FA_); Ba(HMA_JGW_) W(HMA); Bi(HMA_CDW_); Ba(HMA_JGW_) W(HMA); Bi(HMA_PMB_); Ba(HMA_JGW_) W(HMA); Bi(HMA_PMA_); Ba(HMA_JGW_)	4	5	20	22.1	2.6	0.97	0.03	0.29	0.04
W(HMA); Bi(HMA); Ba(CMRA) W(HMA); Bi(HMA_FA_); Ba(CMRA) W(HMA); Bi(HMA_CDW_); Ba(CMRA) W(HMA); Bi(HMA_PMA_); Ba(CMRA) W(HMA); Bi(HMA); Ba(CMRA_JGW_) W(HMA); Bi(HMA_FA_); Ba(CMRA_JGW_) W(HMA); Bi(HMA_CDW_); Ba(CMRA_JGW_) W(HMA); Bi(HMA_PMA_); Ba(CMRA_JGW_)	4	7	20	24.5	3.5	0.96	0.01	0.76	0.06
W(HMA); Bi(HMA_PMB_); Ba(CMRA)	4	5	21	20	–	0.95	–	0.67	–
W(HMA); Bi(HMA_PMB_); Ba(CMRA_JGW_)	4	7	18	19	–	0.96	–	0.70	–

^1^ Wearing course (Mixture ID); Binder layer (Mixture ID); Base layer (Mixture ID).

## Data Availability

Data are contained within the article or supplementary material or are available on request from the corresponding author.

## Supplementary Materials

The following are available online at https://www.mdpi.com/article/10.3390/ma14143867/s1, Table S1: title, Life cycle inventory data sources; Table S2: Life cycle impact category indicators of all the pavement configurations; Table S3: Life cycle impacts of pavement configuration W (HMA), Bi (HMA), Ba (HMA); Table S4: Life cycle impacts of pavement configuration W (HMA), Bi (HMACDW), Ba (HMA); Table S5: Life cycle impacts of pavement configuration W (HMA), Bi (HMAFA), Ba (HMA); Table S6: Life cycle impacts of pavement configuration W (HMA), Bi (HMAPMB), Ba (HMA); Table S7: Life cycle impacts of pavement configuration W (HMA), Bi (HMAPMA), Ba (HMA); Table S8: Life cycle impacts of pavement configuration W (HMA), Bi (HMA), Ba (HMAJGW); Table S9: Life cycle impacts of pavement configuration W (HMA), Bi (HMACDW), Ba (HMAJGW); Table S10: Life cycle impacts of pavement configuration W (HMA), Bi (HMAFA), Ba (HMAJGW); Table S11: Life cycle impacts of pavement configuration W (HMA), Bi (HMAPMB), Ba (HMAJGW); Table S12: Life cycle impacts of pavement configuration W (HMA), Bi (HMAPMA), Ba (HMAJGW); Table S13: Life cycle impacts of pavement configuration W (HMA), Bi (HMA), Ba (CMRA); Table S14: Life cycle impacts of pavement configuration W (HMA), Bi (HMACDW), Ba (CMRA); Table S15: Life cycle impacts of pavement configuration W (HMA), Bi (HMAFA), Ba (CMRA); Table S16: Life cycle impacts of pavement configuration W (HMA), Bi (HMAPMB), Ba (CMRA); Table S17: Life cycle impacts of pavement configuration W (HMA), Bi (HMAPMA), Ba (CMRA); Table S18: Life cycle impacts of pavement configuration W (HMA), Bi (HMA), Ba (CMRAJGW); Table S19: Life cycle impacts of pavement configuration W (HMA), Bi (HMACDW), Ba (CMRAJGW); Table S20: Life cycle impacts of pavement configuration W (HMA), Bi (HMAFA), Ba (CMRAJGW); Table S21: Life cycle impacts of pavement configuration W (HMA), Bi (HMAPMB), Ba (CMRAJGW); Table S22: Life cycle impacts of pavement configuration W (HMA), Bi (HMAPMA), Ba (CMRAJGW).

## Abbreviations

RAP, Reclaimed Asphalt Pavement; CDW, Construction and Demolition Waste; FA, Fly Ash; JGW, Jet Grouting Waste; OBC, Optimum Bitumen Content; HMA, Hot Mix Asphalt manufactured with natural aggregates and neat bitumen; HMA_CDW_, Hot Mix Asphalt manufactured with Construction and Demolition Waste in substitution of natural aggregates; HMA_FA_, Hot Mix Asphalt manufactured with Fly Ash in substitution of natural aggregates; HMA_JGW_, Hot Mix Asphalt manufactured with Jet Grouting Waste in substitution of natural aggregates; HMA_PMB_, Hot Mix Asphalt manufactured with Polymer Modified Bitumen in substitution of natural bitumen; HMA_PMA_, Hot Mix Asphalt manufactured with neat bitumen and recycled polymer pellets for asphalt mixture modification; CMRA, Cold Mix Recycled Asphalt manufactured with Reclaimed Asphalt Pavement in substitution of natural aggregates; CMRA_JGW_, Cold Mix Recycled Asphalt manufactured with Reclaimed Asphalt Pavement and Jet Grouting Waste in substitution of natural aggregates; ITSM, Indirect Tensile Stiffness Modulus; CD, cumulative fatigue damage; RD, rut depth; LCA, Life Cycle Assessment; LCI, Life Cycle Inventory; LCIA, Life Cycle Impact Assessment; GWP, Global Warming Potential, ODP, Ozone Depletion Potential; IR, Ionizing radiation; OFT, damage of ozone formation on terrestrial ecosystems; OFH, damage of ozone formation on human health; PM, fine particulate matter formation; A, terrestrial acidification; FE, freshwater eutrophication, ME, marine eutrophication, T-ECO, terrestrial ecotoxicity; F-ECO, freshwater ecotoxicity; M-ECO, marine ecotoxicity; CT, human carcinogenic toxicity; NCT, human non-carcinogenic toxicity; LU, land use; MR, mineral resource scarcity; FR, fossil resource scarcity; W, water consumption.
